# Investigation Into the Role of Reductants and Cosubstrates in Lytic Polysaccharide Monooxygenase *Thermothielavioides terrestris* AA9E Binding to Cellulose by Single‐Molecule Imaging

**DOI:** 10.1002/bit.70080

**Published:** 2025-10-09

**Authors:** Benedikt M. Blossom, Peter M. Goodwin, Camilla Fløien Angeltveit, Svein Jarle Horn, Alex O. Hitomi, Tina Jeoh

**Affiliations:** ^1^ Department of Chemistry Colby College Waterville Maine USA; ^2^ Department of Geosciences and Natural Resource Management University of Copenhagen Denmark; ^3^ Department of Physics, College of Art and Sciences University of South Florida Tampa Florida USA; ^4^ Center for Integrated Nanotechnologies Los Alamos National Laboratory Los Alamos New Mexico USA; ^5^ Norwegian Environment Agency Norway; ^6^ Faculty of Chemistry, Biotechnology, and Food Science Norwegian University of Life Sciences (NMBU) Ås Norway; ^7^ Department of Biological and Agricultural Engineering University of California Davis Davis California USA

**Keywords:** glucose oxidase/catalase oxygen‐scavenging, lytic polysaccharide monooxygenase (LPMO), protocatechuic acid/protocatechuate 3,4‐dioxygenase oxygen‐scavenging, super‐resolution single‐molecule total internal reflection fluorescence microscopy, *Thermothielavioides terrestris* AA9E (*Tt*AA9E)

## Abstract

Cellulose‐active Lytic Polysaccharide Monooxygenases (LPMO) facilitate plant cell wall deconstruction by attacking ordered regions of cellulose. In vitro, reductants (e.g., ascorbic acid) reduce LPMOs to Cu(I)‐LPMO, and hydrogen peroxide (H_2_O_2_) serves as co‐substrate for oxidative cleavage of cellulose glycosidic bonds. Super‐resolution single‐molecule imaging by total internal reflection fluorescence microscopy was used to visualize and enumerate binding events of fluorescently‐labeled *Thermothielavioides terrestris* AA9E (*Tt*AA9E) on highly ordered cellulose fibrils in oxygen‐scavenging buffer systems. In the glucose oxidase/catalase (GODCAT) system, oxygen is converted to H_2_O_2_, then removed by catalase. Adding ascorbic acid to the GODCAT system promoted rapid binding to cellulose by *Tt*AA9E. In contrast, absent both oxygen and H_2_O_2_ in the protocatechuic acid/protocatechuate 3,4‐dioxygenase (PCA/PCD) oxygen‐scavenging system, adding ascorbic acid nearly eliminated cellulose binding by *Tt*AA9E. Our results suggest that in the GODCAT system, *Tt*AA9Es are reduced by ascorbic acid and activated by H_2_O_2_, facilitating binding to cellulose. In the PCA/PCD system, reduced *Tt*AA9Es are not activated due to the lack of H_2_O_2_, suggesting that reduced Cu(I)‐*Tt*AA9E cannot bind to cellulose without H_2_O_2_. Notably, in the PCA/PCD system with ascorbic acid, oxidized sugar release initially lagged but was observed at longer reaction times, suggesting that H_2_O_2_ could be a limiting reactant generated in situ as oxygen becomes absorbed into solution. Binding durations of LPMO to cellulose were independent of experimental conditions: ( 82% ± 6%) of cellulose‐bound LPMOs resided briefly for 14 ± 2.5 s, while 16% ± 5% of the bound enzymes remained for 60 ± 9 s.

## Graphical abstract

In glucose oxidase + catalase (GODCAT) and protocatechuic acid/protocatechuate 3,4‐dioxygenase (PCA/PCD) oxygen‐scavenging buffers, some Cy5‐*Tt*AA9E bound to crystalline cellulose fibrils. Adding ascorbic acid (AscA) vastly increased enzyme binding to cellulose in the GODCAT buffer but diminished enzyme binding to cellulose in the PCA/PCD buffer.

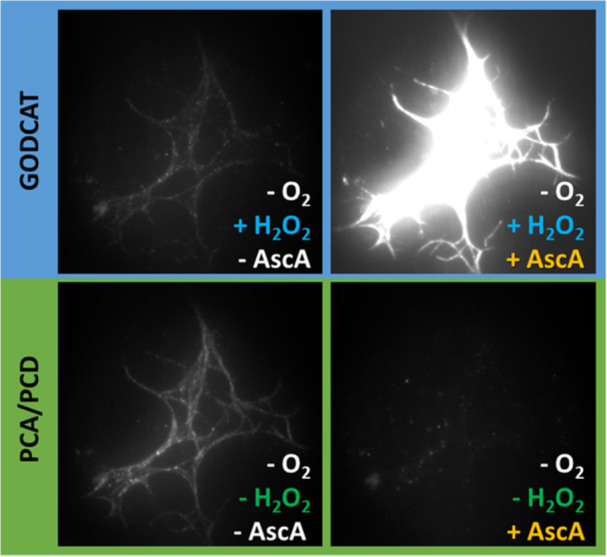

## 1 Introduction

Cellulose fibrils, highly ordered assemblies of linear glucan chains, require a suite of cellulose‐active enzymes, including cellulases and Lytic Polysaccharide Monooxygenases (LPMOs) for complete solubilization to monomeric glucose (Chylenski et al. 2019). Cellulases hydrolyze cellulose by first forming active complexes with single cellulose chains extracted from the fibril surfaces, thus limiting the accessibility of cellulases to disordered regions of cellulose (Jeoh et al. 2024; Karuna and Jeoh 2017; Nill and Jeoh 2020). In contrast, LPMOs, oxidative cellulose‐active enzymes, can cleave glycosidic bonds in ordered regions of cellulose without needing to complex with an isolated and solvent‐accessible cellulose chain (Horn et al. 2012). LPMOs are suggested to play a critical role in creating surface disorder to increase solvent accessibility in ordered cellulose (Uchiyama et al. 2022) that can increase the availability of productive cellulase binding sites (Angeltveit et al. 2023). Thus, understanding conditions that promote productive LPMO‐cellulose interactions are vital to maximize the effectiveness of LPMOs in cellulose degradation.

LPMOs are non‐hydrolytic “accessory” members of polysaccharide degrading enzymes produced by many saprophytic bacteria and fungi. The “monooxygenase” moniker stems from initial speculation that these enzymes utilized molecular oxygen to fuel oxidative cleavage reactions of glycosidic bonds in cellulose (Vaaje‐Kolstad et al. 2010). However, more recently, LPMOs have come to be viewed as peroxygenases because hydrogen peroxide (H_2_O_2_) fueled reactions have been shown to be significantly more efficient than oxygen‐fueled reactions (Bissaro et al. 2017). While many have studied reaction conditions that facilitate oxidative activity of LPMOs on cellulose, few have specifically examined conditions necessary for productive LPMO‐cellulose binding. Cellulose‐active LPMOs are metalloenzymes that harness copper to oxidatively cleave cellulose glycosidic bonds (Horn et al. 2012). Cellulose‐active LPMOs produced by fungi (AA9) and bacteria (AA10) share a similar flat active site structure wherein a copper ion is held by a histidine brace in the catalytic center of the enzyme (Levasseur et al. 2013; Quinlan et al. 2011). The consensus mechanism for LPMO activity on cellulose is that the priming reduction of Cu(II) to Cu(I) in the LPMO active site precedes oxidative cleavage of cellulose glycosidic bonds where either the C1 or C4 carbon of the glucose residues become oxidized (Bissaro and Eijsink 2023). Reduction of the copper can be mediated by small molecule reductants such as ascorbic acid or gallic acid (Wang et al. 2020), lignin (Dimarogona et al. 2012), and other redox‐active enzymes (Kracher et al. 2016). While LPMO reactions work with either oxygen or hydrogen peroxide, studies have shown that the latter cosubstrate results in faster reaction rates by several orders of magnitude (Bissaro et al. 2017; Kuusk and Väljamäe 2021). Furthermore, H_2_O_2_ is produced in situ in systems with oxygen and reductants, suggesting that H_2_O_2_ is the relevant co‐substrate. LPMOs presumably bind to flat, ordered, hydrophobic surfaces of cellulose fibrils where the proximity of the catalytic center to glycosidic bonds facilitate catalysis (Zhou et al. 2020). Studies have shown that both the affinity and binding extents of LPMO on microcrystalline cellulose (MCC) and phosphoric acid swollen cellulose (PASC) increase in reactions with a reductant such as ascorbic acid in both aerobic and anoxic reactions (Kracher et al. 2018). Moreover, the residence times of LPMO on the surface of cellulose, measured by high‐speed atomic force microscopy, was reported as ~1 min (Eibinger et al. 2017). While the need to reduce Cu(II) to Cu(I) in the LPMOs for oxidative cleavage of cellulose is clear, an open question is whether binding of the cosubstrate H_2_O_2_ in the active site must precede productive binding of the LPMOs to cellulose.

**Figure 1:**
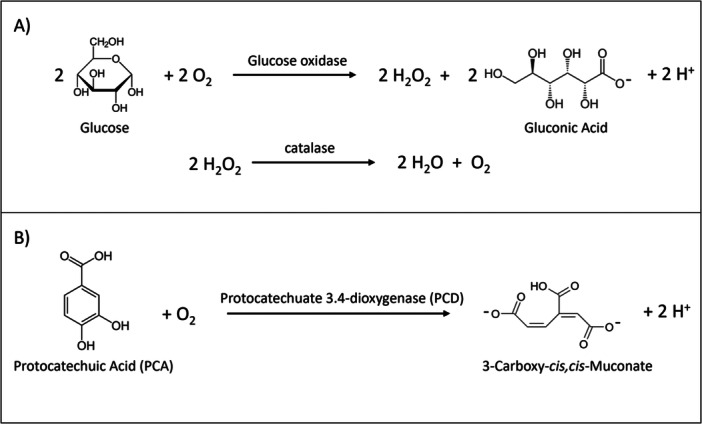
The mechanisms for removal of dissolved oxygen by (A) the glucose oxidase + catalase (GODCAT) oxygen‐scavenging system; and (B) the PCA/PCD oxygen‐scavenging system.

Super‐resolution single‐molecule imaging by total internal reflection fluorescence microscopy (TIRFM) is a powerful tool for time‐resolved visualization of the dynamics of individual enzymes binding to the surface of cellulose (Haviland et al. 2021, 2024; Jung et al. 2013; Mudinoor et al. 2020; Nousi et al. 2024). Single‐molecule TIRFM imaging allows simultaneous quantification of hundreds to thousands of binding events and binding durations under varying aqueous reaction conditions. Single‐molecule TIRFM imaging is typically conducted under anoxic conditions to prevent premature quenching of the fluorophores. A commonly used oxygen‐scavenging system is the glucose oxidase (GOx) and catalase system, herein referred to as the GODCAT system, where oxygen is consumed when GOx oxidizes glucose to gluconic acid (Figure 1A). The GODCAT system generates H_2_O_2_ as an intermediate that is further scavenged by the catalase. The GODCAT system has been successfully employed to increase the photostability of the Cy5 fluorophore under laser excitation (Aitken et al. 2008), to measure extended residence times of cellulase binding to cellulose (Haviland et al. 2021; Jung et al. 2013; Mudinoor et al. 2020). An alternate oxygen‐scavenging buffer system employs protocatechuate 3,4‐dioxygenase (PCD) that consumes oxygen when oxidizing protocatechuic acid (PCA) (Figure 1B). This PCA/PCD oxygen‐scavenging system has been shown to deplete oxygen more rapidly, and to a greater extent than the GODCAT system (Aitken et al. 2008). Of relevance to experiments with LPMOs, the PCA/PCD system does not generate H_2_O_2_, and the product of oxidation, 3‐carboxy‐*cis, cis*‐muconic acid, can chelate metals. Most studies on LPMO reactions in the literature are conducted under “monooxygenase conditions”, that is, aerobic with a small molecule reductant (i.e., no direct addition of H_2_O_2_ in the reactions, but likely leading to in situ H_2_O_2_ generation). The experiments in this study were conducted under oxygen‐limited conditions maintained either by the GODCAT or the PCA/PCD oxygen‐scavenging buffers.

In this study, we examined conditions that promote cellulose binding by LPMO using fluorescently‐labeled *Thermothielavioides terrestris* AA9E (*Tt*AA9E), a C1‐oxidizing, unimodular (i.e., without a cellulose binding module) LPMO (Kadowaki et al. 2021; Kim et al. 2017; Tõlgo et al. 2022) on highly ordered algal cellulose fibrils (O'Dell et al. 2015). Single‐molecule TIRFM experiments were conducted in the GODCAT and the PCA/PCD oxygen‐scavenging systems that modulated the availability of oxygen and hydrogen peroxide, allowing an examination of how the cosubstrate impacts *Tt*AA9E binding to cellulose. The role of small molecular weight reductants on LPMO‐cellulose interactions was examined using ascorbic acid and gallic acid in the two oxygen‐scavenging systems. Finally, the importance of copper in LPMO‐cellulose binding was tested using holo‐ and apo‐LPMO with and without additional metal chelation. Individual Cy5‐labeled *Tt*AA9E binding events and binding durations were measured under the various reaction conditions to understand individual and combined contributions of O_2_/H_2_O_2_ cosubstrate, external reductant, and copper availability on substrate binding. Moreover, activity experiments under similar reaction conditions were conducted to determine the productive or nonproductive nature of the observed LPMO‐cellulose binding outcomes.

## 2 Materials and Methods

### 2.1 Lytic Polysaccharide Monooxygenase (LPMO) Enzyme Preparation

Lytic Polysaccharide Monooxygenase (LPMO) enzyme *Tt*AA9E from *Thermothielavioides terrestris* was kindly gifted from Novonesis (Novonesis Inc., Bagsværd, Denmark). The LPMO was copper saturated as described previously (Loose et al. 2014). In brief, the LPMO was incubated in a 3:1 molar ratio with Cu(II)SO_4_ before the excess copper was removed by desalting using a PD MidiTrap column (G‐25; GE Healthcare, Chicago, IL, USA). *Tt*AA9E was covalently labeled with Cyanine 5 (Cy5) fluorophores as described previously (Jung et al. 2013; Mudinoor et al. 2020), resulting in a degree of labeling of 0.85 dye/protein (mole/mole). Apo‐LPMO was prepared by extensive incubation in EDTA at a ratio of 1 mM EDTA per 2 µM LPMO. All LPMOs were stored at 4°C when not in use.

### 2.2 Cellulose Fibrils Preparation

Algal cellulose fibrils were purified from *Cladophora aegagropila* and treated with concentrated hydrochloric acid as previously described (Mudinoor et al. 2020). The never‐dried cellulose fibrils with lengths on the order of 10 µm and thickness ranging from ~5–20 nm were deposited onto hydrophobically‐silanized glass coverslips by gravity‐aided settling and assembled into imaging channels as previously described (Jung et al. 2013; Mudinoor et al. 2020). Before use, unbound cellulose was rinsed off with buffer and the surfaces were passivated by incubating with 10 mg/mL of Bovine Serum Albumin (BSA) for > 10 min.

### 2.3 *Tt*AA9E Binding to Cellulose Under Varying Reaction Conditions

Various experiments examining the impact of oxygen‐scavenging buffers, pH, and reductants on Cy5‐labeled *Tt*AA9E binding to cellulose were conducted. The glucose oxidase–catalase (GODCAT) oxygen‐scavenging system consisted of 0.2 U/µL glucose oxidase (Avantor Science Central), 2 U/µL catalase (Avantor Science Central), 2% (w/v) glucose (Sigma‐Aldrich), and Trolox (6‐hydroxy‐2,5,7,8‐tetramethylchroman‐2‐carboxylic acid) (Sigma‐Aldrich) in 50 mM sodium acetate buffer at pH 5 or phosphate buffer at pH 7.5. The protocatechuic acid/protocatechuate‐3,4‐dioxygenase (PCA/PCD) oxygen‐scavenging system consisted of 2.5 mM PCA (Sigma‐Aldrich) and 50 nM PCD (Sigma‐Aldrich) in 50 mM phosphate buffer at pH 7.5. The PCA/PCD oxygen‐scavenging system is unstable at pH 5.

A typical experiment was conducted at room temperature, where the Cy5‐labeled enzymes at a concentration of 50–100 pM in an oxygen‐scavenging buffer were loaded into the 10 μL channel immediately before imaging. In select experiments, 1 or 2 mM of ascorbic acid or gallic acid was added as a reductant. In experiments with apo‐LPMO, 10 mM EDTA was also included with the oxygen‐scavenging buffer.

### 2.4 Single‐Molecule Imaging of Individual Cy5‐Labeled Enzymes by Total Internal Reflection Fluorescence Microscopy (TIRFM)

A detailed description of the through‐objective TIRFM setup has been previously described in (Jung et al. 2013; Mudinoor et al. 2020). Briefly, single‐molecule fluorescence images of individual Cy5‐labeled enzyme molecules binding and unbinding from cellulose fibrils were imaged using 637 nm laser excitation reflected by a multiline dichroic mirror (FF500/646‐Di01, Semrock) and focused at the back aperture of a 1.49 NA 60× oil‐immersion objective (Olympus) to provide total‐internal‐reflection (TIR) excitation at the cover glass/water interface across a ~50 µm diameter area in the object plane. Sample emission was collected and imaged by the same objective onto the 512 × 512‐pixel sensor of an electron multiplying CCD (EMCCD) camera (Photonmax, Princeton Instruments). A 37 nm wide bandpass filter centered at 676 nm was used to isolate Cy5 fluorescence excited at 637 nm. For the binding time measurements, the overall magnification of the imaging system (73×) mapped each EMCCD pixel to a 220 × 220 nm area in the object plane. Relatively low power (~0.2 mW @ 637 nm) excitation was used to reduce the effect of Cy5 photobleaching on the binding time measurements. Under similar imaging conditions, the time to bleach of most (90%) of Cy5 fluorophores exhibited an extended characteristic decay time of 1101 s in the GODCAT oxygen‐scavenging buffer system (Supporting Information S1). Image stacks were collected at EMCCD camera integration times of 1 s.

### 2.5 Binding Time Analysis

Binding times of individual enzymes in the imaging experiments were determined as previously described (Mudinoor et al. 2020). Briefly, centroid x–y coordinates of Cy5‐labeled enzymes within a masked area of each image in the image stacks were located using the DAOSTORM algorithm (Holden et al. 2011) and compiled into binding time histograms with a custom algorithm. Multiexponential fitting with the differential evolution (genetic optimization) algorithm (Wormington et al. 1999) and minimized using Poisson deviance cost function (Steinbach 2012) implemented in Igor Pro (Wavemetrics, ver. 6.34 A) determined characteristic binding times.

### 2.6 *Tt*AA9E Activity on Cellulose in GODCAT or PCA/PCD Oxygen‐Scavenging Buffers

LPMO activity in the oxygen‐scavenging systems (GODCAT or PCA/PCD) was tested by incubating 0.25% (w/v) algal cellulose in a 96‐well plate with 0.5 µM *Tt*AA9E and a reaction volume of 300 µL at 25°C, 600 rpm for 30 min. If added, ascorbic acid (Sigma‐Aldrich) was present at 1 mM. The oxygen‐scavenging buffers were prepared as described above in a potassium phosphate buffer (50 mM, pH 7.5; Sigma‐Aldrich); however, they were without the addition of Trolox. Both buffer systems were incubated for 30 min before *Tt*AA9E was added. The oxygen‐scavenging effect of the buffers was confirmed by performing a colorimetric test with resazurin sodium salt (Sigma‐Aldrich) (data not shown). The reactions were stopped at different time points by filtering using a 96‐well filter plate (Sigma‐Aldrich).

LPMO activity was confirmed with ascorbic acid and PCA as reductants under aerobic conditions in separate experiments (Supporting Information S1).

### 2.7 Soluble Sugar Analysis With High‐Performance Anion‐Exchange Chromatography

The soluble LPMO products were analyzed by high‐performance anion‐exchange chromatography with pulsed amperometric detection (HPAEC‐PAD) using a Dionex ICS‐5000 (Thermo Scientific). A CarboPac PA1 column with an eluent gradient from 0% to 100% B (A: 100 mM NaOH; B: 1 M NaOAc + 100 mM NaOH), an operational flow of 500 µL/min, and a sample loop volume of 5 µL was used (Østby et al. 2023; Westereng et al. 2013). The samples were treated with an endoglucanase from *Thermobifida fusca* (2 µM, *Tf*Cel6A; produced in‐house, as described previously (Spezio, et al. 1993) to quantify the amount of soluble sugars released.

C1‐oxidized standards, GlcGlc1A and Glc2Glc1A, were made by treating cellobiose or cellotriose, respectively, with a cellobiose dehydrogenase from *Myriococcum thermophilum* (0.2 µM, *M*tCDH; produced in‐house, as described previously (Zámocký et al. 2008). The reactions were performed in sodium acetate buffer (50 mM, pH 5.0) at 40°C for 20 h in an Eppendorf Thermomixer (Eppendorf, Hamburg, Germany).

## 3 Results

### 3.1 *Tt*AA9E Binding to Cellulose in the GODCAT Oxygen‐Scavenging System at pH 5 Is Impacted by Different Reductants

**Figure 2:**
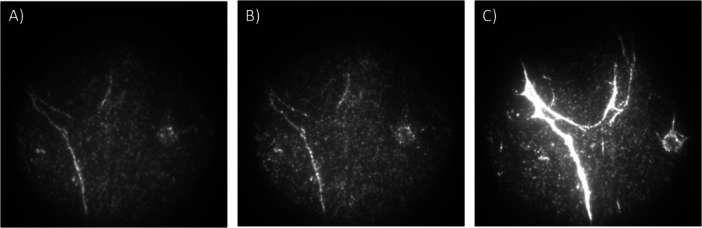
Visualizing the impact of different reductants on *Tt*AA9E binding to cellulose in the same 31.8 × 31.8 µm field of view (FOV). (A) in the GODCAT buffer system that includes Trolox intended to suppress fluorophore blinking (Supporting Information S2: Video SV1), (B) after the subsequent addition of 1 mM gallic acid (Supporting Information S3: Video SV2) to the system in (A), and (C) after the subsequent addition of 2 mM ascorbic acid to the system in (B) (Supporting Information S4: Video SV3) at pH 5. The images are averaged intensity of (A) 1509, (B) 507, and (C) 108 sequential frames collected at 1 fps. Brightness and contrast are matched across the three images to facilitate visual comparison.

Super‐resolution single‐molecule imaging using Cy5‐labeled enzymes is best conducted in oxygen‐free environments to increase the photostability of the Cy5 label under 637 nm laser excitation (Aitken et al. 2008). The glucose oxidase–catalase (GODCAT) oxygen system, used effectively in the study of Cy5‐labeled Cel7A binding to cellulose (Mudinoor et al. 2020), scavenges dissolved oxygen by oxidizing glucose and includes a catalase to remove the resulting H_2_O_2_ coproduct (Figure 1) (Aitken et al. 2008). Trolox, a water‐soluble form of vitamin E, was included with the GODCAT buffer system to minimize fluorophore blinking, a phenomenon where fluorophores intermittently go dark, which complicates measurements of residence times of the labeled enzymes (Aitken et al. 2008). In our experiments at pH 5, we observed some binding of *Tt*AA9Es to cellulose fibrils in the GODCAT buffer, suggesting that the antioxidant Trolox could serve as an electron donor to *Tt*AA9E (Figure 2A). Adding gallic acid to the system in GODCAT buffer only marginally improved *Tt*AA9E binding to cellulose, suggesting that under these conditions (pH 5), gallic acid was not significantly better than Trolox at promoting *Tt*AA9E binding to cellulose (Figure 2B). The addition of ascorbic acid, however, vastly increased *Tt*AA9E binding to cellulose, as clearly visualized by the intense fluorescence of the cellulose fibrils attributable to LPMO binding (Figure 2C). The experiment in Figure 2 was conducted by sequential observation first only in the GODCAT buffer (Figure 2A, Supporting Information S2: Video SV1), followed by adding gallic acid (Figure 2B, Supporting Information S3: Video SV2), then ascorbic acid (Figure 2C, Supporting Information S4: Video SV3). LPMO binding to cellulose in the GODCAT buffer was initially sparse, and built to higher concentration (Supporting Information S2: Video SV1), but upon adding ascorbic acid, the increase in fluorescence intensity of the cellulose fibrils in the field of view was immediate.

Trolox, gallic acid, and ascorbic acid have the potential to act as reductants for LPMOs under aerobic and anaerobic environments (Bissaro et al. 2017; Kracher et al. 2018, 2016; Kuusk et al. 2019). In this case, of the three reductants, ascorbic acid was the most effective at promoting rapid *Tt*AA9E binding to cellulose. Previous studies have shown that oxidized LPMOs can bind to cellulose in anaerobic conditions, albeit at reduced affinity and extents (Kracher et al. 2018). Thus, the slow buildup of LPMO binding to cellulose could suggest that under these conditions, neither Trolox noraA gallic acid were effective reductants for the LPMOs. In the given reaction condition of minimal oxygen at pH 5, it appears that ascorbic acid was most effective at rapidly priming *Tt*AA9E, leading to enhanced binding to cellulose.

### 3.2 Copper Is Necessary for *Tt*AA9E Binding to Cellulose

**Figure 3:**
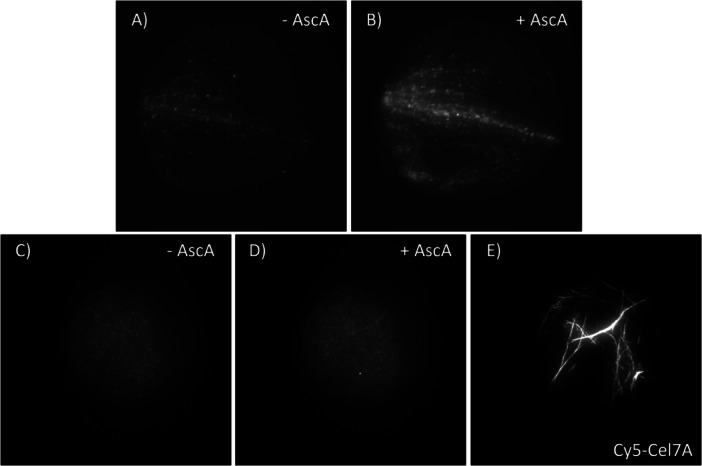
Binding of apo‐*Tt*AA9E to cellulose at pH5 in GODCAT oxygen‐scavenging buffer + 10 mM EDTA without ascorbic acid (Supporting Information S5: Video SV4) (A) and with 1 mM ascorbic acid (Supporting Information S6: Video SV5) (B). Binding of apo‐*Tt*AA9E incubated overnight in 10 mM EDTA to cellulose in GODCAT oxygen‐scavenging buffer + 10 mM EDTA without ascorbic acid (Supporting Information S7: Video SV6) (C) and with 1 mM ascorbic acid (Supporting Information S8: Video SV7) (D) (10× higher enzyme concentration than in (A) and (B)). The presence of cellulose fibrils in the (C) and (D) FOV was verified by adding Cy5‐Cel7A (Supporting Information S9: Video SV8) (E). (A and B) are of the same FOV, and (C–E) are of the same FOV. Images are of the averaged intensity of an image stack; brightness and contrast are matched for all images shown. Dimensions of all FOVs shown are 31.8 × 31.8 µm.

The importance of active site copper in *Tt*AA9E for binding to cellulose was examined using apo‐*Tt*AA9E. In initial experiments, despite removing ~95% of copper from the *Tt*AA9E enzyme preparation and the presence of 10 mM EDTA in the reaction, some *Tt*AA9E binding to cellulose in the GODCAT buffer could be seen and further enhanced by the addition of ascorbic acid (Figure 3A,B). In this case, the presence of free copper in solution is unlikely; thus, the increase in LPMO binding to cellulose upon addition of ascorbic acid is most likely due to the reduction of the ~5% of holoenzymes by ascorbic acid.

Suspecting that the cellulose‐bound *Tt*AA9Es are likely attributable to residual holoenzymes, the *Tt*AA9E preparation was incubated overnight in 10 mM EDTA before repeating the binding experiments. Indeed, apo‐*Tt*AA9E incubated overnight in EDTA showed almost no binding to cellulose in the GODCAT buffer without ascorbic acid, despite using 10‐fold higher enzyme concentration (Figure 3C). However, even after overnight incubation in EDTA and the presence of EDTA in the reaction, the faintest trace of fluorescently lit cellulose fibrils was observed when ascorbic acid was added, indicating some enzyme binding to cellulose (Figure 3D). In agreement with previous studies (Aachmann et al. 2012; Kracher et al. 2018; Quinlan et al. 2011), we conclude from these observations that *Tt*AA9E has a very strong affinity to copper such that complete copper removal is nontrivial, even using a vast excess of a strong chelator such as EDTA. Furthermore, removing copper indeed disables all binding of the LPMOs to cellulose. The presence of cellulose in the field of view with apo‐*Tt*AA9E was verified by the subsequent addition of Cy5‐Cel7A, previously demonstrated to bind specifically to cellulose fibrils under similar imaging conditions (Jung et al. 2013; Mudinoor et al. 2020) (Figure 3E).

### 3.3 Comparing *Tt*AA9E Binding to Cellulose in GODCAT and PCA/PCD Oxygen‐Scavenging Buffers at pH 7.5

The GODCAT buffer system, intended to scavenge dissolved oxygen that could compromise the stability and longevity of the Cy5‐dye in the imaging experiments, generates H_2_O_2_ as an intermediate to facilitate cellulose oxidation (Wang et al. 2020). While catalase in the GODCAT buffer system removes the hydrogen peroxide, the low affinity of catalase (*K*_*m,*H2O2_ on the order of 100 mM (Switala and Loewen 2002)) and high affinity of LPMOs (*K*_*m,*H2O2_ in the micromolar range (Bissaro et al. 2017; Kuusk et al. 2023; Kuusk and Väljamäe 2021)) means that LPMOs can compete for H_2_O_2_ at low concentrations in the GODCAT buffer system. The binding experiments were conducted in an alternate oxygen‐scavenging buffer system using protocatechuic acid (PCA)/protocatechuate‐3,4‐dioxygenase (PCD). Unlike the GODCAT system, the PCA/PCD system utilizes oxygen without generating hydrogen peroxide (Aitken et al. 2008) (Figure 1B), thus allowing further exploration as to whether depleting the cosubstrates (O_2_ and H_2_O_2_) would impact binding to cellulose. Moreover, Aitken et al. (2008) found the PCA/PCD system to reduce dissolved oxygen concentrations at a faster rate and to a greater extent than the GODCAT system. Of note, the PCA oxidation product, 3‐carboxy‐*cis, cis*‐muconic acid (3‐CMA), can chelate metals including copper. A higher pH of 7.5 was necessary for comparing the two oxygen‐scavenging buffer systems because the PCA/PCD system is unstable at pH 5. A side‐by‐side comparison of variables of the two oxygen‐scavenging buffer systems that potentially impact LPMO activity is shown in Table 1.

**Table 1:** Side‐by‐side comparison of the GODCAT and PCA/PCD buffer systems used in the experiments. The oxygen‐scavenging reactions are shown in Figure 1.

	GODCAT system	PCA/PCD system
O_2_ removal	Yes	Yes
H_2_O_2_ generation	Yes (scavenged by catalase)	No
Copper chelation	No	Yes (3‐carboxy‐*cis, cis*‐muconic acid)
Potential LPMO reductant	Trolox	PCA

**Figure 4:**
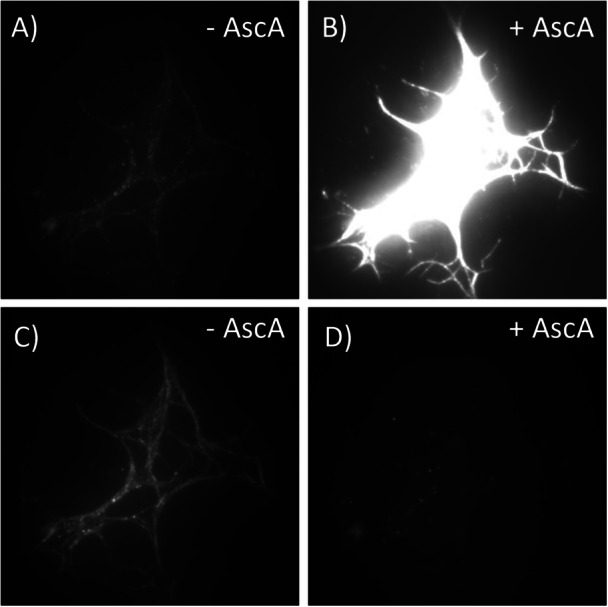
Visualizing *Tt*AA9E binding to cellulose in GODCAT buffer at pH 7.5 (A and B, Supporting Information S10, S11: Videos SV9 and SV10) and in PCA/PCD buffer at pH 7.5 (C and D, Supporting Information S12, S13: Videos SV11 and SV12), without ascorbic acid (A and C) and with 1 mM ascorbic acid (B and D). All four images were taken in the same FOV using the same enzyme concentrations. Images are of the averaged intensity of an image stack; brightness and contrast are matched for all images shown. Dimensions of all FOVs shown are 31.8 × 31.8 µm.

**Figure 5:**
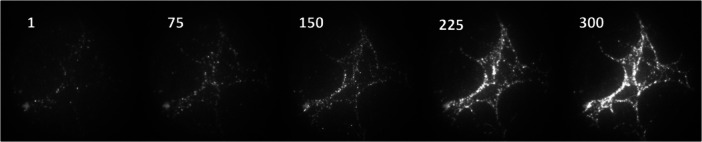
*Tt*AA9E binding to cellulose in PCA/PCD buffer at pH 7.5 over 5 min (Supporting Information S14: Video SV13). Select frames from an image stack of 307 frames taken at 1 fps are shown. Frame numbers (also corresponding to imaged time in seconds) are indicated in each image. Images are of averaged intensity of an image stack; brightness and contrast are matched for all images shown. Dimensions of all FOVs shown are 31.8 × 31.8 µm.

While we had already observed *Tt*AA9E binding to cellulose in the GODCAT buffer at pH 5 (Figure 2A), *Tt*AA9E binding to cellulose was also observed in the GODCAT buffer at pH 7.5 (Figure 4A), and in the PCA/PCD buffer system at pH 7.5 (Figure 4C). As observed with *Tt*AA9E binding to cellulose in the GODCAT buffer (Supporting Information S2: Video SV1), *Tt*AA9E binding to cellulose in the PCA/PCD system was initially sparse and built up over time (Figure 5 and Supporting Information S14: Video SV13). The slow buildup of *Tt*AA9E binding to cellulose suggests that oxidized *Tt*AA9E (Cu(II)‐*Tt*AA9E) can bind to cellulose, albeit with lower affinity as observed by the slow buildup, in agreement with previous observations (Kracher et al. 2018).

Upon addition of ascorbic acid, however, *Tt*AA9E exhibited opposite cellulose binding behaviors in the two oxygen‐scavenging systems. In the GODCAT system, ascorbic acid addition vastly enhanced *Tt*AA9E binding to cellulose as evidenced by an increase in fluorescence intensity exceeding saturation (Figure 4B, Supporting Information S11: Video SV10). In contrast, adding ascorbic acid to the PCA/PCD system vastly diminished *Tt*AA9E binding to cellulose, almost completely extinguishing the fluorescence signal (Figure 4D, Supporting Information S13: Video SV12). The increase in *Tt*AA9E binding to cellulose upon adding ascorbic acid in the GODCAT system was an expected result, as was already observed at pH 5 (Figures 2, 3). The dramatic decrease in *Tt*AA9E binding to cellulose in the PCA/PCD system when ascorbic acid was added, however, was a surprise. Ascorbic acid has only a slight impact on fluorophore stability (Aitken et al. 2008), thus, the loss in fluorescence observed in Figure 4D is not likely due to fluorescence quenching, but rather a direct impact on LPMO binding to cellulose.

### 3.4 Characteristic Binding Times of *Tt*AA9E on Cellulose

**Figure 6:**
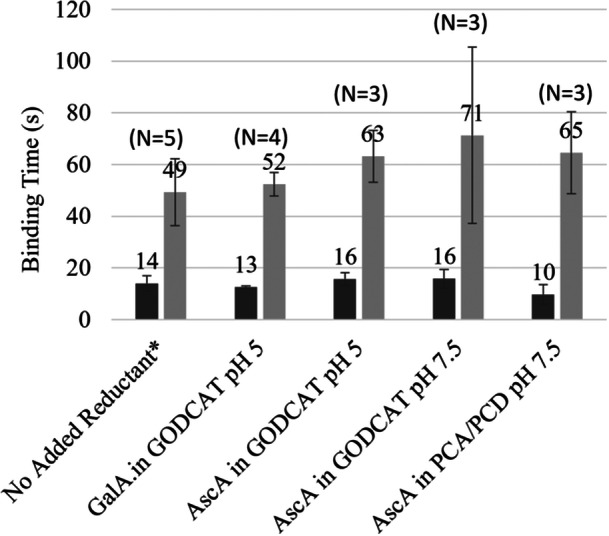
Characteristic binding times of *Tt*AA9E on cellulose measured in PCA/PCD (pH 7.5) or GODCAT buffers (pH 5 and 7.5) with ascorbic acid (AscA) or gallic acid (GalA). Two populations with short (dark gray) and long (light gray) characteristic binding times are shown. *In the case where no reductant was added, residence times were averaged regardless of buffer or pH used in the experiment. Averages from multiple (*N*) experiments are shown.

In a previous study, the binding time of LPMO on cellulose of ~1 min was estimated by high‐speed AFM (Eibinger et al. 2017). A major strength of super‐resolution single‐molecule imaging lies in compiling binding times of thousands of individual enzymes, from which characteristic binding time distributions can be extracted by multiexponential fitting with a high degree of confidence. A 3‐exponential decay model best fit the binding time distributions of *Tt*AA9Es on cellulose in all experiments conducted in this study. Overall, regardless of the oxygen‐scavenging system, solution pH, reductant type or availability, LPMOs bound to cellulose exhibited similar characteristic binding times (Figure 6). Overall averages of all experiments indicated that most of the bound enzymes ( 82% ± 6%) remained bound only briefly for 14 ± 2.5 s, while 16% ± 5% of the enzymes remained bound for 60 ± 9 s. A small 1%–2% fraction of the bound enzymes appeared to bind for an extended time; however, assessment of the longer binding times was prevented by photobleaching of the Cy5 label. The predominance of short‐duration binding events (~14 s) is consistent with previous measurements of *Tr*Cel7A binding to algal cellulose (Mudinoor et al. 2020). These brief interactions are likely nonspecific binding events and fall below the time‐resolution threshold high‐speed AFM detection, hence not previously noted.

### 3.5 Soluble Sugar Production From *Tt*AA9E Acting on Cellulose in GODCAT and PCA/PCD Oxygen‐Scavenging Buffers

**Figure 7:**
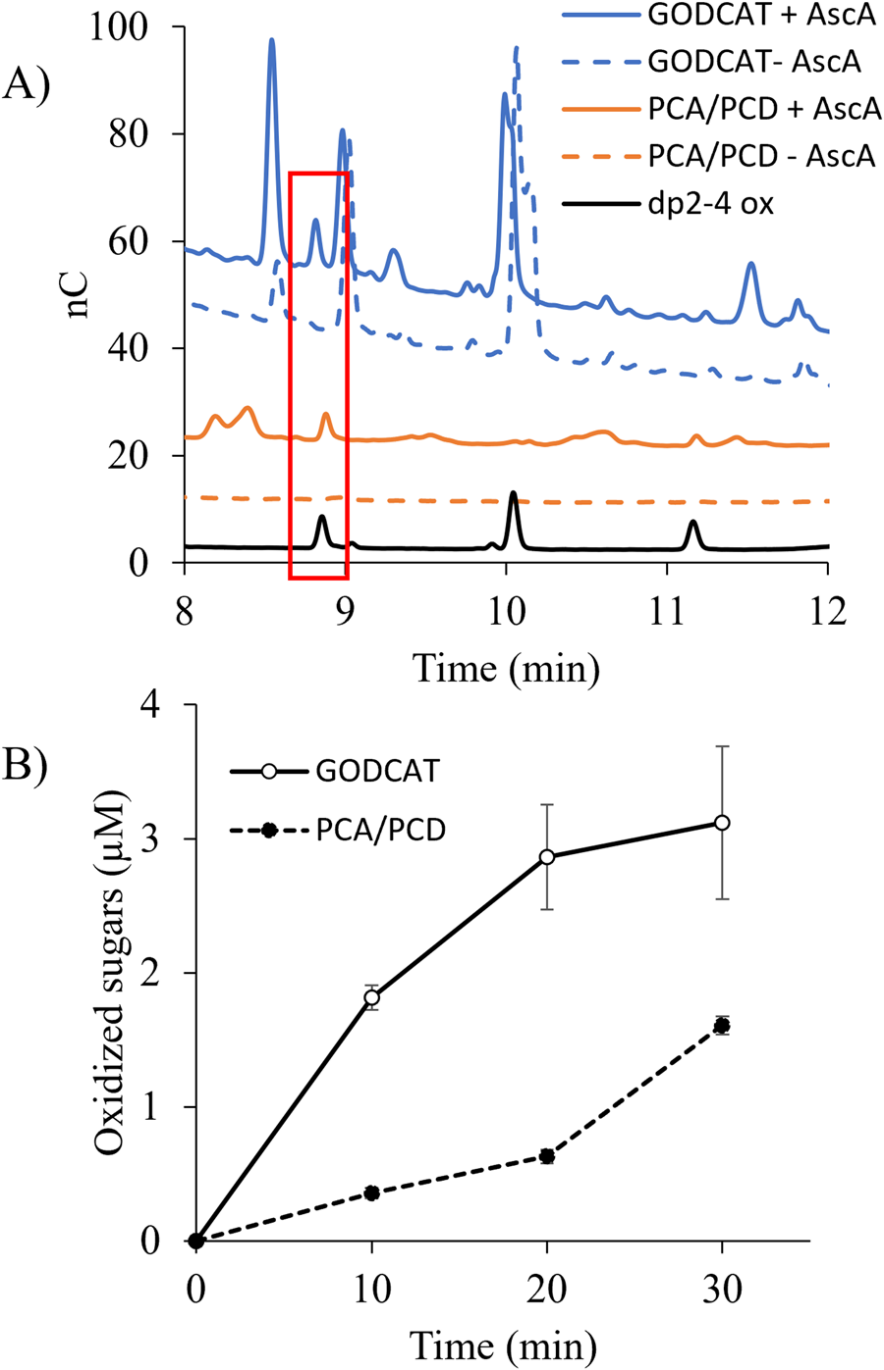
(A) Stacked chromatograms demonstrating oxidized sugar production by *Tt*AA9E acting on Avicel with ascorbic acid (+AscA, solid lines), but not without ascorbic acid (−AscA, dashed lines) in the GODCAT and PCA/PCD oxygen‐scavenging systems. Samples after 30 min of reaction were treated with *Tf*Cel6A. Red box highlights oxidized disaccharide (dp2 ox) peaks. Oxidized sugar standards (dp2‐4 ox) are shown in black. (B) Time course of oxidized sugars released by *Tt*AA9E using ascorbic acid in reactions with algal cellulose in either the GODCAT or PCA/PCD oxygen‐scavenging system. Lines are drawn only to guide the eye.

The single‐molecule imaging experimental setup, while allowing direct assessment of substrate binding, was unfortunately not compatible with measuring whether binding was associated with oxidative cleavage of cellulose by the LPMOs. Thus, separate experiments were conducted to measure any release of soluble oxidized sugars. Initially, it was confirmed that both ascorbic acid and PCA could act as electron donors for *Tt*AA9A under aerobic conditions (Supporting Information S1: S1.3). In both the GODCAT and PCA/PCD oxygen‐scavenging buffer systems, without ascorbic acid, no soluble sugar release attributable to the LPMO action on algal cellulose was detected. However, with the addition of ascorbic acid, oxidized sugars were detected in LPMO reactions with Avicel, a microcrystalline cellulose, in both buffer systems after 30 min of incubation (Figure 7A). The time course of oxidized sugar release from algal cellulose showed that oxidized sugar production in the GODCAT system was initially rapid and decelerated over time reaching a maximum around 3 µM (Figure 7B). In contrast, oxidized sugar production in the PCA/PCD system exhibited a delayed onset, which accelerated with time. This trend suggests that a limiting reaction component was initially scarce but became increasingly available. In the PCA/PCD buffer, H_2_O_2_ is the likely limiting reactant; moreover, the activity experiments conducted in unsealed 96‐well plates may have allowed oxygen absorption over time, facilitating H_2_O_2_ generation in the presence of ascorbic acid. The single‐molecule imaging experiments conducted in the PCA/PCD buffer system (Figure 4C,D) were only over 5‐min periods, during which the data in Figure 7 suggests there was minimal enzyme activity.

## 4 Discussion

To carry out oxidative cleavage of polysaccharides, LPMOs require externally sourced electrons that are transferred via a mediator (Wang et al. 2020). Although initially thought to be a monooxygenase sourcing electrons from molecular oxygen, recent studies have driven the consensus that LPMOs are more likely peroxygenases that use hydrogen peroxide as the preferred co‐substrate (Bissaro et al. 2017; Kont et al. 2020; Kuusk and Väljamäe 2021). As such, LPMO activity has been demonstrated in anoxic reaction conditions supplied with hydrogen peroxide and an electron mediator such as ascorbic acid (Kracher et al. 2018). In this study, owing to the need to maintain fluorophore stability for prolonged imaging of fluorescently‐labeled LPMOs, oxygen‐scavenging buffers were used with the intent to significantly reduce the dissolved oxygen content in the reactions. The two oxygen‐scavenging systems used in this study, the GODCAT and PCA/PCD systems, had a secondary benefit of modulating oxygen and hydrogen peroxide availability in the reactions, providing interesting insights into the role of cosubstrates in LPMO binding to cellulose.

**Figure 8:**
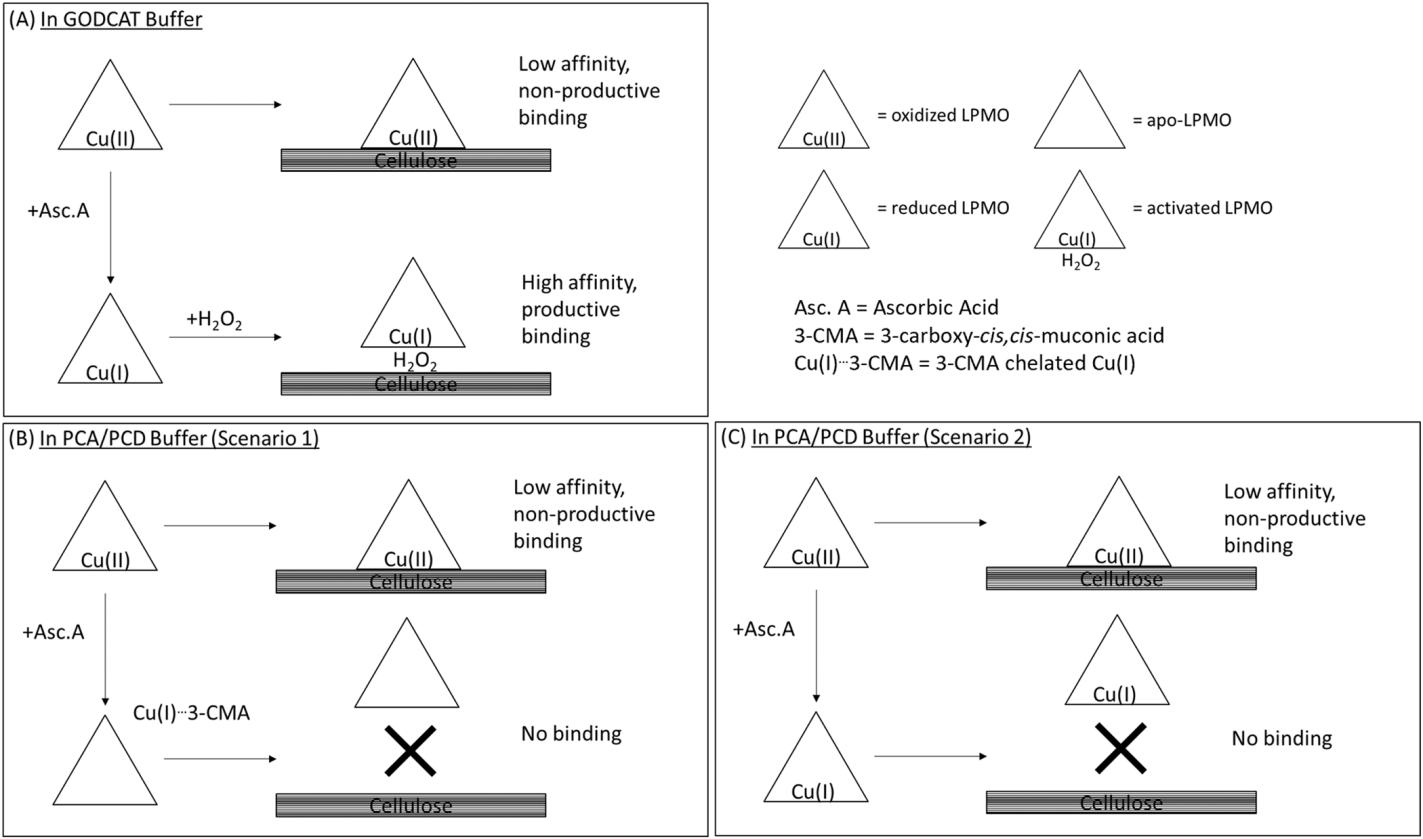
Schematics of hypothetical mechanisms of (A) low and high affinity binding to cellulose by oxidized, and activated LPMO, respectively, in the GODCAT buffer, (B) low affinity binding to cellulose by oxidized LPMO, and no binding due to loss and chelation of Cu(I) from the LPMO when ascorbic acid is added to the PCA/PCD buffer, and (C) low affinity binding to cellulose by oxidized LPMO and no binding by reduced, but not activated LPMO.

The GODCAT system, intended to reduce the dissolved oxygen content, generates hydrogen peroxide that can be utilized by LPMOs with ~1000‐fold greater affinity than the catalases intended to remove H_2_O_2_ (Bissaro et al. 2017; Kuusk et al. 2023; Kuusk and Väljamäe 2021; Switala and Loewen 2002). In reactions in the GODCAT system, visibly lower extents of binding of the LPMOs to cellulose was observed with Trolox and gallic acid as the only potential reductants. Only the addition of ascorbic acid visibly and significantly enhanced LPMO binding to cellulose (Figure 8A). Kracher et al. demonstrated that oxidized LPMOs can bind to cellulose but with lower affinity and to a decreased extent (Kracher et al. 2018). As also seen by others (Bissaro et al. 2017; Kracher et al. 2018), oxidized sugar concentrations in GODCAT buffered reactions without ascorbic acid were below detection, suggesting negligible LPMO activity on cellulose in these reactions. It appears, therefore, that under the imaging conditions used, neither Trolox nor gallic acid were effective reductants, and that LPMOs that do bind to cellulose without first being reduced are likely not productively bound.

Reactions in the PCA/PCD oxygen‐scavenging system gave interesting insights. When both PCA and PCD are present, oxygen is rapidly consumed. Unlike the GODCAT system, the PCA/PCD system does not generate hydrogen peroxide (Figure 1). Even though PCA could serve as a reductant for LPMO (Supporting Information S1: S1.3), with no oxygen and no hydrogen peroxide, the PCA/PCD buffer system offers no cosubstrate to drive substrate oxidation. Thus, supported by the absence of oxidized sugar production in the PCA/PCD buffer, the observed LPMO binding to cellulose was likely nonproductively bound LPMOs. But why did *Tt*AA9E binding to cellulose visibly diminish when ascorbic acid was added into the PCA/PCD buffered reaction? Here, we consider two possible explanations for the observed loss of *Tt*AA9E binding to cellulose upon addition of ascorbic acid in the PCA/PCD buffer. In the first scenario (Figure 8B), ascorbic acid reduces *Tt*AA9E‐Cu(II) to *Tt*AA9E‐Cu(I), but the Cu(I) is subsequently chelated by 3‐CMA, the product of the PCA/PCD reaction, leaving apo‐*Tt*AA9E that does not bind to cellulose. This scenario is unlikely because 3‐CMA is, at best, a weak chelator for Cu(II) and poorly suited for Cu(I) chelation. Moreover, as demonstrated in Figure 3, *Tt*AA9E exhibit exceptionally strong copper binding affinity, surpassing even EDTA, a well‐established strong copper chelator. Notably, near complete removal of copper from ~100 pM of *Tt*AA9E required extensive incubation in 10 mM EDTA, underscoring the remarkable chelation strength of *Tt*AA9Es. Given that 3‐CMA is a weak chelator, it is improbable that it could strip Cu(I) from a reduced *Tt*AA9E.

A second possible explanation for the loss of *Tt*AA9E binding to cellulose in the PCA/PCA buffer when ascorbic acid was added is that reduced *Tt*AA9E, without a cosubstrate, cannot bind cellulose (Figure 8C). The notion that reduced LPMOs must first bind to H_2_O_2_ before binding to cellulose is highly controversial and contradicts conventional belief. It is commonly assumed that reduced LPMOs bind to the substrate before H₂O₂, based on evidence that substrate binding protects LPMOs from oxidative damage, since reactions with H₂O₂ in the absence of substrate can lead to off‐pathway inactivation (Hegnar et al. 2019; Paradisi et al. 2019; Torbjörnsson et al. 2023). Due to the challenges in precisely controlling both oxygen and H_2_O_2_ concentrations in reactions, there is currently no direct evidence in the literature establishing binding order of reduced LPMO to substrate versus H_2_O_2_ (Eijsink et al. 2019). Nonetheless, cosubstrate binding before substrate by LPMOs is not implausible, as shown by a > 1000‐fold enhancement in an AA9 LPMO binding to substrate in the presence of chloride ions (Frandsen et al. 2016). In this study, single‐molecule imaging provided a unique means to directly visualize how reaction components influence *Tt*AA9E binding to cellulose. We present evidence strongly suggesting that in the absence of both oxygen and H_2_O_2_, addition of ascorbic acid that reduces *Tt*AA9E results in a loss of LPMO binding to cellulose. Thus, our experiments identify at least two conditions that prevent *Tt*AA9E binding to cellulose: copper depletion (apo‐LPMO), and reduction without access to a cosubstrate.

The time course of oxidized sugar release in Figure 7B showed minimal LPMO activity in the PCA/PCD system with ascorbic acid, likely due to the lack of H_2_O_2_, which is consistent with previous studies confirming the necessity of H_2_O_2_ for hydroxylation of cellulose (Bissaro et al. 2017). However, Figure 7B also showed acceleration of LPMO activity with time, suggesting that H_2_O_2_ could be generated in the reaction over time. As the activity assays were conducted in unsealed well‐plates, oxygen can absorb in the reaction leading to H_2_O_2_ generation. The necessity of reducing LPMOs from Cu(II) to Cu(I) for activity on polysaccharides has been clearly established from the early days of LPMO research (Phillips et al. 2011; Quinlan et al. 2011). However, this study suggests that reduction (i.e., priming of the LPMO) alone does not promote binding, and activation by H_2_O_2_ binding to the reduced LPMO may precede specific and productive substrate binding.

It is interesting to note that the binding durations of LPMOs on cellulose were similar in all reaction conditions. The amount of time that LPMOs spend on the surface of cellulose, therefore, appears to be unrelated to whether the enzyme is active. This situation is similar to that of the exocellulase Cel7A, where studies have shown that the duration that Cel7A remains complexed to cellulose is limited by the interactions between cellulose and the amino acid residues in the active site that are not coupled to hydrolysis time (Kari et al. 2014). Given that enzymatic deconstruction of cellulose is an interfacial reaction on a substrate where many enzymes compete for access to the limited surface area, one potential LPMO research direction is to focus on engineering amino acids around the active site that are responsible for substrate binding to reduce non‐productive occupation of precious real estate on cellulose.

## 5 Conclusions

By visualizing single‐molecule LPMO‐cellulose binding events under anoxic reaction environments with and without in situ H_2_O_2_, we observed that reduction of the LPMOs (i.e., the priming step) alone may be insufficient for specific and productive binding. Productive binding of LPMO to cellulose may in fact require both an effective reductant to prime and a cosubstrate like H_2_O_2_ to activate the LPMOs. Our study underscores the need for further continued investigation into the binding order of co‐substrate and substrate by LPMOs.

Although some cellulose binding was observed in the absence of either reductant or cosubstrate, this binding was not associated with detectable soluble sugar production, that is, most likely non‐productive binding. Regardless of whether under priming‐promoting or non‐promoting conditions, ~82% of the LPMOs at the surface of cellulose resided for ~14 s, while ~16% resided for ~60 s. The residence times of LPMOs on the surface of cellulose appeared to be unrelated to whether the enzyme was active.

## Author Contributions

**Benedikt M. Blossom:** conceptualization, methodology, investigation, writing – review and editing, funding acquisition. **Peter M. Goodwin:** methodology, investigation, formal analysis, writing – review and editing, **Camilla Fløien Angeltveit:** investigation, formal analysis. **Svein Jarle Horn:** formal analysis, writing – review and editing. **Alex O. Hitomi :** investigation, writing – review and editing, **Tina Jeoh:** data curation, formal analysis, investigation, methodology, project administration, validation, visualization, writing – original draft, review, and editing.

## Conflicts of Interest

The authors declare no conflicts of interest.

## Supporting information

Supporting information 1: S1.1 Photostability of Cy5 in the glucose oxidase/catalase (GODCAT) buffer system. S1.2 Summary of imaging conditions. S1.3 Activity of *Tt*AA9E using Ascorbic Acid (AscA) or Protocatechuic Acid (PCA) as reductant in aerobic reactions. Supporting videos compressed to jpg format at 7 frames per second (7fps) are provided here. All videos are of Cy5‐labeled *Tt*AA9E with algal cellulose fibrils unless otherwise specified; buffer and additive information provided in the filename. Enzyme, substrate, and image acquisition details are provided in the Experimental Procedures section.

SV1 TtAA9E GODCAT pH 5 no reductant.

SV2 TtAA9E GODCAT pH 5 Gallic Acid.

SV3 TtAA9E GODCAT pH 5 Ascorbic Acid.

SV4 apo*Tt*AA9E GODCAT pH 5 no reductant + 10 mM EDTA.

SV5 apo*Tt*AA9E GODCAT pH 5 Ascorbic Acid + 10 mM EDTA.

SV6 apoTtAA9E 10X overnight in EDTA GODCAT pH 5 10 mM EDTA.

SV7 apoTtAA9E 10X ON in EDTA GODCAT pH 5 AscA 10 mM EDTA.

SV8 Cel7A GODCAT pH 5 + 10 mM EDTA.

SV9 TtAA9E GODCAT pH 7.5 no reductant.

SV10 TtAA9E GODCAT pH 7.5 + Ascorbic Acid.

SV11 TtAA9E PCA/PCD pH 7.5 no reductant.

SV12 TtAA9E PCA/PCD pH 7.5 + Ascorbic Acid.

SV13 TtAA9E PCA/PCD pH 7.5 no reductant.

## Data Availability

Raw image datafiles corresponding to the jpg‐compressed supporting videos are available at the Dryad database at https://doi.org/10.5061/dryad.9ghx3ffrh.
