# Supplementary material for: Investigation Into the Role of Reductants and Cosubstrates in Lytic Polysaccharide Monooxygenase Thermothielavioides terrestris AA9E Binding to Cellulose by Single‐Molecule Imaging
Source: Biotechnol Bioeng. 2025 Oct 9;123(1):52–63. doi: 10.1002/bit.70080 (PMC12699106; doi:10.1002/bit.70080)
Supplement: Supplementary file 1 — Supporting information 1: S1.1 Photostability of Cy5 in the glucose oxidase/catalase (GODCAT) buffer system. S1.2 Summary of imaging conditions. S1.3 Activity of TtAA9E using Ascorbic Acid (AscA) or Protocatechuic Acid (PCA) as reductant in aerobic reactions. Supporting videos compressed to jpg format at 7 frames per second (7fps) are provided here. All videos are of Cy5‐labeled TtAA9E with algal cellulose fibrils unless otherwise specified; buffer and additive information provided in the filename. Enzyme, substrate, and image acquisition details are provided in the Experimental Procedures section. [file BIT-123-52-s005.docx]

**Supplementary Information 1**

Investigation into the role of reductants and cosubstrates in lytic polysaccharide monooxygenase *Thermothielavioides terrestris* AA9E binding to cellulose by single molecule imaging

Benedikt M. Blossom^1,†^, Peter M. Goodwin^2,‡^, Camilla Fløien Angeltveit^3,§^, Svein Jarle Horn^3^, Alex Hitomi^4^, Tina Jeoh^4,*^

^1^ Department of Geosciences and Natural Resource Management, University of Copenhagen, DK-1958 Frederiksberg C, Denmark.

^2^ Center for Integrated Nanotechnologies, Los Alamos National Laboratory, Los Alamos, NM, 87545, USA.

^3^ Faculty of Chemistry, Biotechnology, and Food Science, Norwegian University of Life Sciences (NMBU), Ås 1433, Norway

^4^ Department of Biological and Agricultural Engineering, University of California Davis, Davis, California, USA.

† Current: Colby College, Department of Chemistry, Waterville, Maine, USA.

‡ Current: University of South Florida, Department of Physics, College of Art and Sciences, Tampa, Florida, USA.

§ Current: Norwegian Environmental Agency

^*^Corresponding author: [tjeoh@ucdavis.edu](mailto:tjeoh@ucdavis.edu)

# Photostability of Cy5 in the glucose oxidase/catalase (GODCAT) buffer system

The photostability of the Cy5 label was measured at pH 5 with and without the GODCAT buffer system under the imaging setup and conditions used in this study (see main manuscript). Cy5-labeled streptavidin was immobilized onto a biotinylated surface and excited at 637 nm and 0.2 mW; total Cy5 fluorescence intensities were measured over time (Figure S1. 1). Consistent with past experiments conducted similarly ^1^, the fluorescence signal could be modeled as a biexponential decay. The fluorophores exhibited an extended fluorescence lifetime in the GODCAT buffer system where 90% had a characteristic decay lifetime of 1100 s (Table S1.1).


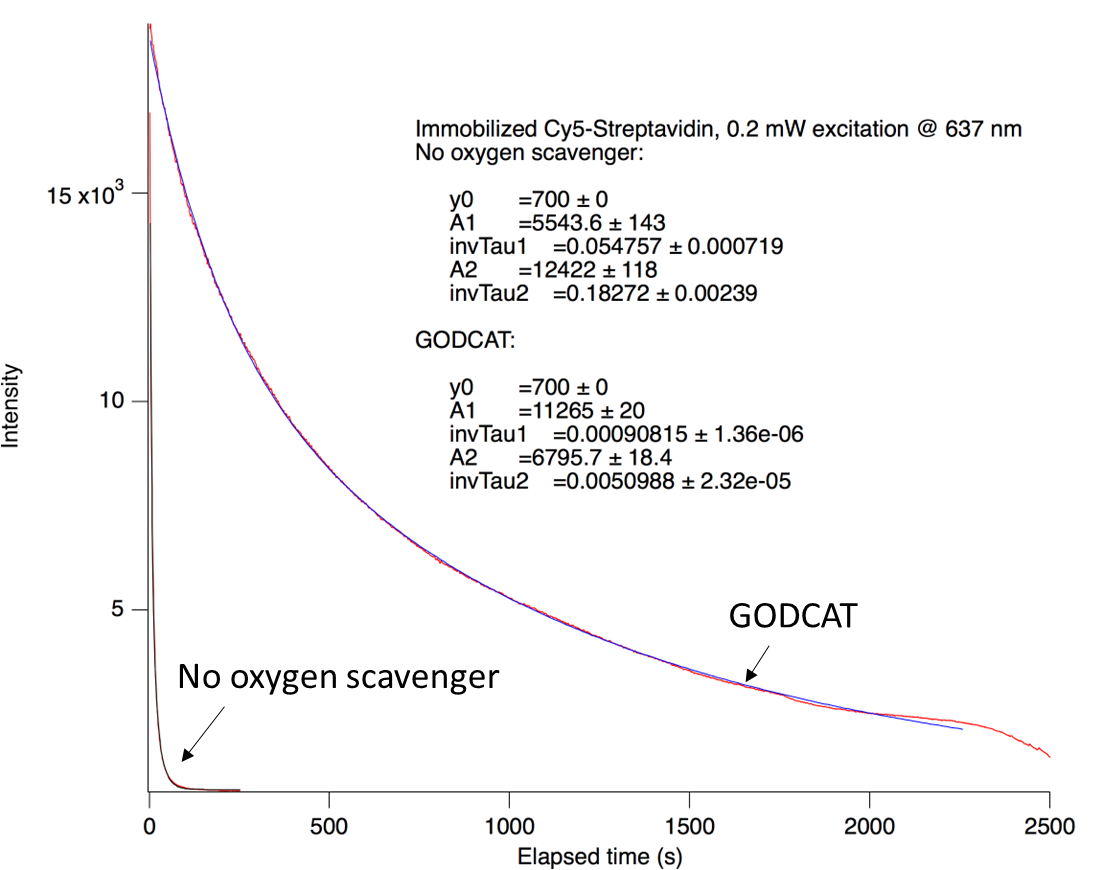


Figure S1. 1: Fluorescence intensity of Cy5-labeled streptavidin measured over time in sodium acetate pH 5 with and without the GODCAT oxygen scavenging buffer system.

Table S1. 1: Total Cy5 fluorescence decay times with and without the GODCAT oxygen scavenging system calculated from biexponential fits to data shown in Figure S1. 1.

|  | POPULATION 1  % of Total / Decay Time (s) | POPULATION 2  % of Total / Decay Time (s) |
| --- | --- | --- |
| - oxygen scavenging buffer | 40 % / 5.5 s | 60 % / 18.3 s |
| + GODCAT | 10 % / 196 s | 90 % / 1101 s |

# Summary of imaging conditions

Table S1. 2 compares imaging conditions, buffer components and binding outcomes observed in this study. The only conditions that resulted in no detectable binding were when >95% apo*Tt*AA9E were used, and when ascorbic acid was added to the reaction in PCA/PCD buffer (shown in red box).

Table S1. 2: Summary of imaging conditions and resulting binding outcomes

| Oxygen Scavenging Buffer System | GODCAT | | | | | | | | | PCA/PCD | |  |
| --- | --- | --- | --- | --- | --- | --- | --- | --- | --- | --- | --- | --- |
| Enzyme | *Tt*AA9E | *Tt*AA9E | *Tt*AA9E | 95% apo  *Tt*AA9E | 95% apo  *Tt*AA9E | >95% Apo  *Tt*AA9E | >95% Apo  *Tt*AA9E | *Tt*AA9E | *Tt*AA9E | *Tt*AA9E | *Tt*AA9E | |
| pH | 5 | | | | | | | 7.5 | | | |  |
| H_2_O_2_ | + | + | + | + | + | + | + | + | + | - | - | |
| Reductant | Trolox | Trolox  Gallic Acid | Trolox  Asc. Acid | Trolox | Trolox  Asc. Acid | Trolox | Trolox  Asc. Acid | Trolox | Trolox  Asc. Acid | PCA | PCA  Asc. Acid | |
| Chelator | - | - | - | EDTA | EDTA | EDTA | EDTA | - | - | 3-CMA | 3-CMA | |
| Specific Binding | + | + | +++ | Trace | + | - | - | + | ++++ | + | - | |
| Figure | 2A | 2B | 2C | 3A | 3B | 3C | 3D | 4A | 4B | 4C | 4D | |
| Video | SV1 | SV2 | SV3 | SV4 | SV5 | SV6 | SV7 | SV9 | SV10 | SV11 | SV12 | |

*3-CMA: 3-carboxy-cis,cis-muconic acid*

# Activity of *Tt*AA9E using Ascorbic Acid (AscA) or Protocatechuic Acid (PCA) as reductant in aerobic reactions

The effectiveness of ascorbic acid (AscA) and protocatechuic acid (PCA) as external reductants in aerobic reactions were tested using algal cellulose (AC) and Avicel. *Tt*AA9E was incubated with either AscA (1 mM) or PCA (2.5 mM or 12.5 mM) in 50 mM potassium phosphate buffer, pH. 7.5. *Tt*AA9E activity on AC and Avicel with AscA could be seen by the presence of peaks at D1ox and D2ox (Figure S1. 2). The presence of PCA resulted in a high baseline in the chromatograms (Figure S1. 2A and B, such that the presence of oxidized sugars in the hydrolysates were difficult to detect when AC was used as a substrate. However, oxidized sugar peaks were clearly visible in the reactions with PCA on Avicel. Increased concentrations of PCA appeared to have minimal impact on the abundance of oxidized sugar release as seen as similar-sized peaks in the chromatograms. Similarly, the addition of free copper did not appear to impact activity. These tests, therefore, confirmed that both AscA and PCA can act as electron donors for *Tt*AA9E under aerobic conditions.


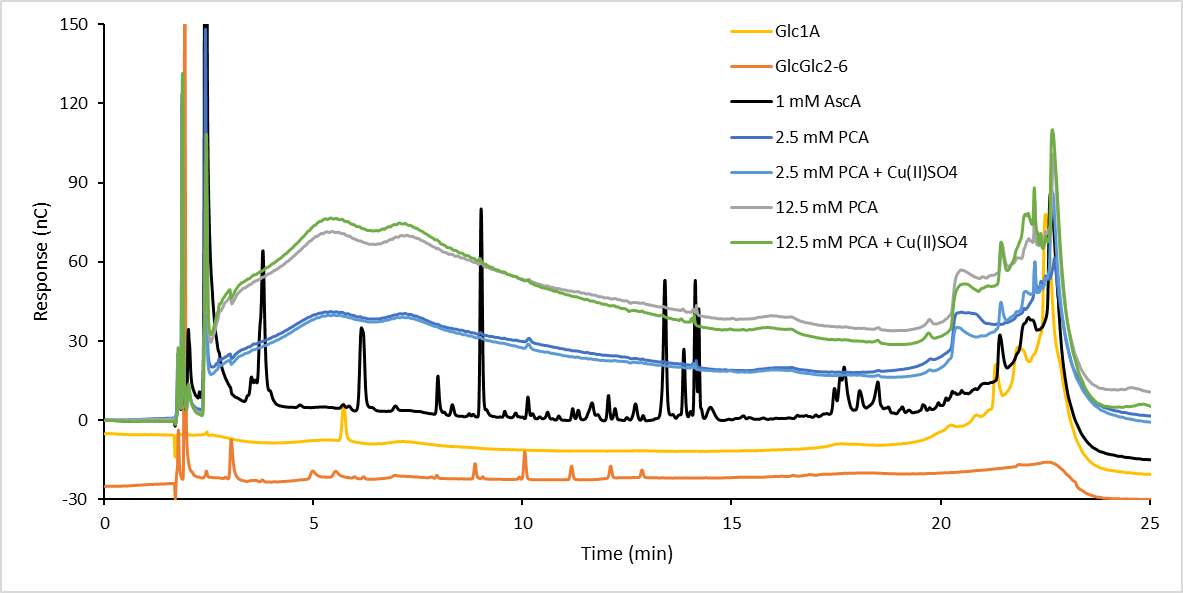

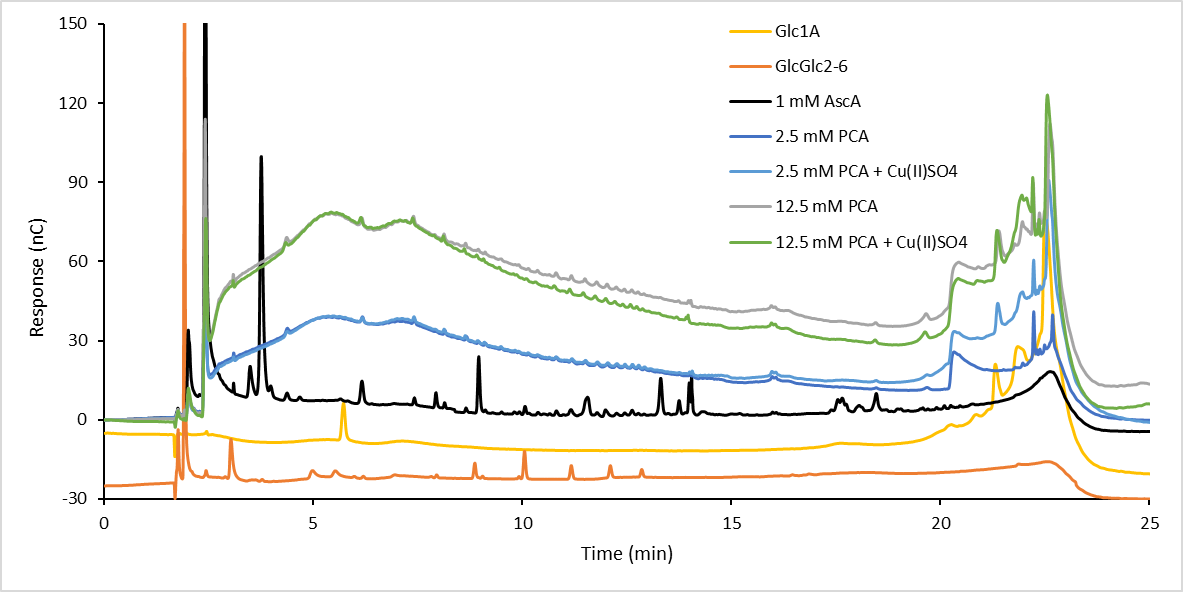


B)

A)

Figure S1. 2: (A) Chromatograms of hydrolysates obtained after 1 hour incubation with **algal cellulose** using 1 mM Ascorbic Acid, 2.5 mM PCA, 2.5 mM PCA + Cu(II)SO_4_, 12.5 mM PCA, or 12.5 mM PCA + Cu(II)SO_4_; (B) Chromatograms of hydrolysates obtained after 1 hour incubation with **Avicel** using 1 mM Ascorbic Acid, 2.5 mM PCA, 2.5 mM PCA + Cu(II)SO4, 12.5 mM PCA, or 12.5 mM PCA + Cu(II)SO4.

# References

(1) Mudinoor, A. R.; Goodwin, P. M.; Rao, R. U.; Karuna, N.; Hitomi, A.; Nill, J.; Jeoh, T. Interfacial Molecular Interactions of Cellobiohydrolase Cel7A and Its Variants on Cellulose. *Biotechnol. Biofuels* **2020**, *13* (1), 10. https://doi.org/10.1186/s13068-020-1649-7.
